# Prediction of fall events during admission using eXtreme gradient boosting: a comparative validation study

**DOI:** 10.1038/s41598-020-73776-9

**Published:** 2020-10-08

**Authors:** Yin-Chen Hsu, Hsu-Huei Weng, Chiu-Ya Kuo, Tsui-Ping Chu, Yuan-Hsiung Tsai

**Affiliations:** 1 Department of Diagnostic Radiology, Chang Gung Memorial Hospital Chiayi Branch, Chiayi, Taiwan; 2 College of Medicine, Chang Gung University, Taoyuan, Taiwan; 3 Department of Nursing, Chang Gung Memorial Hospital Chiayi Branch, Chiayi, Taiwan

**Keywords:** Computational biology and bioinformatics, Health care, Risk factors

## Abstract

As the performance of current fall risk assessment tools is limited, clinicians face significant challenges in identifying patients at risk of falling. This study proposes an automatic fall risk prediction model based on eXtreme gradient boosting (XGB), using a data-driven approach to the standardized medical records. This study analyzed a cohort of 639 participants (297 fall patients and 342 controls) from Chang Gung Memorial Hospital, Chiayi Branch, Taiwan. A derivation cohort of 507 participants (257 fall patients and 250 controls) was collected for constructing the prediction model using the XGB algorithm. A comparative validation of XGB and the Morse Fall Scale (MFS) was conducted with a prospective cohort of 132 participants (40 fall patients and 92 controls). The areas under the curves (AUCs) of the receiver operating characteristic (ROC) curves were used to compare the prediction models. This machine learning method provided a higher sensitivity than the standard method for fall risk stratification. In addition, the most important predictors found (*Department of Neuro-Rehabilitation, Department of Surgery, cardiovascular medication use, admission from the Emergency Department, and bed rest*) provided new information on in-hospital fall event prediction and the identification of patients with a high fall risk.

## Introduction

Patient falls are a major issue in health care institutions throughout the world. Despite considerable research in this field, inpatient falls continue to be a common adverse event in clinical practice^1^. In Taiwan, the Department of Health introduced the Taiwan Patient-Safety Reporting (TPR) system in 2003. According to the TPR annual report of 2017, there were 17,104 inpatient falls, accounting for 25.2% of all inpatient safety events, of which 51.9% resulted in injuries^2^. A review of the literature further showed that among the patients who fell during hospitalization, 28% had minor injuries, 11.4% had severe soft tissue injuries, 5% had bone fractures, and approximately 2% had head trauma, which could lead to long-term disability or premature mortality^3^.

To reduce the incidence of inpatient falls, the Joint Commission for Healthcare Organization Accreditation suggested integrating a standardized, validated tool into the medical record system to identify those with a high fall risk^4^. Several studies have been conducted to investigate fall risk factors and develop fall risk assessment tools to aid in the recognition of patients at greater risk of falling. However, the review studies concluded that there is no explicit superiority for any single assessment tool, and no tool correctly identifies fallers with high validity and reliability^5,6^.

eXtreme gradient boosting (XGB), a machine learning system for tree boosting, is widely used by data scientists to achieve state-of-the-art results on many machine learning challenges. Using an XGB algorithm, this study proposed an automatic assessment tool to accurately detect high-risk groups. Other machine learning approaches have been attempted for identifying patients at risk of falling; however, a comparative validation with current fall risk assessment scales has never been involved^7^.

The Morse Fall Scale (MFS), which was developed in 1989, is the most widely used tool for the assessment of fall risk in the United States^8^. This study aims to propose a machine learning model for fall risk assessment in competition with the MFS. We further explore determinants for predicting inpatient fall events.

## Results

### Demographics and clinical characteristics

The study cohort included 639 adult patients (297 fall patients and 342 controls) who were admitted to our institution between February 2015 and December 2018. A derivation cohort of 507 participants (257 fall patients and 250 controls) was collected through June 2018 to develop the prediction model. Between July and December 2018, a validation cohort of 132 participants (40 fall patients and 92 controls) was prospectively collected. Despite the adoption of identical inclusion criteria, there were three items that showed significant differences in demographic features between the derivation and validation cohorts. The full demographic and clinical descriptions of both groups are presented in Table 1.

**Table 1:** Clinical descriptors of the derivation and validation cohorts.

Characteristics		Derivation cohort	Validation cohort	*P* ^b^
		(N = 507)	(N = 132)	
Sex^a^	Male, n (%)	283 (55.82%)	81 (61.36%)	0.253
	Female, n (%)	224 (44.18%)	51 (38.64%)	
Age (y)^a^		69.30 ± 13.74	69.32 ± 15.18	0.988
Admission day	Weekday, n (%)	419 (82.64%)	99 (75.00%)	0.046
	Weekend, n (%)	88 (17.36%)	33 (25.00%)	
Source	Emergency Department, n (%)	294 (57.99%)	78 (59.09%)	0.820
	Outpatient Department, n (%)	213 (42.01%)	54 (40.91%)	
Department	Internal Medicine, n (%)	197 (38.86%)	55 (41.67%)	0.557
	Surgery, n (%)	138 (27.22%)	37 (28.03%)	0.853
	Intensive Care Unit, n (%)	27 (5.32%)	7 (5.30%)	0.993
	Neuro-Rehabilitation, n (%)	139 (27.42%)	31 (23.48%)	0.362
	Other, n (%)	6 (1.18%)	2 (1.52%)	0.754
Ward type	Single room, n (%)	64 (12.62%)	17 (12.88%)	0.936
	Multiperson room, n (%)	443 (87.38%)	115 (87.12%)	
Vital signs^a^	Body temperature (°C)	36.53 ± 1.38	36.34 ± 0.59	0.123
	Pulse rate (/min)	83.78 ± 15.41	81.74 ± 15.14	0.174
	Respiratory rate (/min)	18.88 ± 2.18	18.96 ± 2.29	0.710
	Systolic blood pressure (mmHg)	139.25 ± 63.47	137.39 ± 23.88	0.741
Glasgow coma scale ^a^	14.41 ± 1.95	14.36 ± 2.01	0.794	
Muscle power^a^	Left-upper limb	4.55 ± 1.03	4.62 ± 0.86	0.473
	Right-upper limb	4.57 ± 1.05	4.61 ± 0.84	0.686
	Left-lower limb	4.42 ± 1.07	4.39 ± 1.04	0.773
	Right-lower limb	4.38 ± 1.19	4.42 ± 1.03	0.724
Activity^a^	Normal, n (%)	249 (49.11%)	61 (46.21%)	0.553
	Weak, n (%)	207 (40.83%)	60 (45.46%)	0.337
	Bed rest, n (%)	51 (10.06%)	11 (8.33%)	0.550
Education level^a^	High school or less, n (%)	466 (91.91%)	119 (90.15%)	0.518
	College or above, n (%)	41 (8.09%)	13 (9.85%)	
Marital status^a^	Single, n (%)	42 (8.28%)	8 (6.06%)	0.398
	Married, n (%)	421 (83.04%)	111 (84.09%)	0.774
	Widowed or divorced, n (%)	44 (8.68%)	13 (9.85%)	0.675
Caregiver^a^	None, n (%)	5 (0.99%)	1 (0.76%)	0.808
	Family, n (%)	464 (91.52%)	124 (93.94%)	0.361
	Nursing worker, n (%)	38 (7.49%)	7 (5.30%)	0.381
Ambulatory aid^a^	Free, n (%)	476 (93.89%)	121 (91.67%)	0.360
	Cane or walker, n (%)	20 (3.94%)	4 (3.03%)	0.624
	Wheelchair, n (%)	11 (2.17%)	7 (5.30%)	0.053
FRID^a^	Cardiovascular drugs, n (%)	113 (22.29%)	40 (30.30%)	0.055
	Antidiabetic drugs, n (%)	48 (9.47%)	23 (17.42%)	0.010
	CNS drugs, n (%)	19 (3.75%)	11 (8.33%)	0.027

The values are presented as the mean ± SD, or n (%).

CNS, central nervous system; FRID, fall risk-increasing drugs.

^a^The generalizable factors include intrinsic factors and predictors directly linked to the cause of a fall.

^b^The* P*-values indicate a significant difference between the two cohorts. The* P*-value of means and proportions are obtained by t-test and chi-square test, respectively.

According to the two fall risk assessment tools, the average scores for the fall group and the nonfall group are shown in Table 2. There were significant differences between the fall and nonfall groups in the fall risk assessment tools (*P* < 0.01, *P* < 0.001, and *P* < 0.001, respectively).

**Table 2:** Mean scores and contingency table according to the prediction models (n = 132).

Model	MFS			XGB			XGB-GF		
No fall	33.21 ± 16.38			0.4121 ± 0.2152			0.4099 ± 0.2273		
Fall	41.63 ± 17.77			0.5899 ± 0.2791			0.5852 ± 0.2802		
*p*	< 0.01			< 0.001			< 0.001		

	No	Yes	Sum	No	Yes	Sum	No	Yes	Sum
No fall	64	28	92	69	23	92	64	28	92
Fall	20	20	40	14	26	40	15	25	40
Sum	84	48	132	83	49	132	79	53	132

XGB-GF, the XGB model using generalizable factors including intrinsic factors and predictors directly linked to the cause of a fall.

### Performance of the prediction models

**Figure 1:**
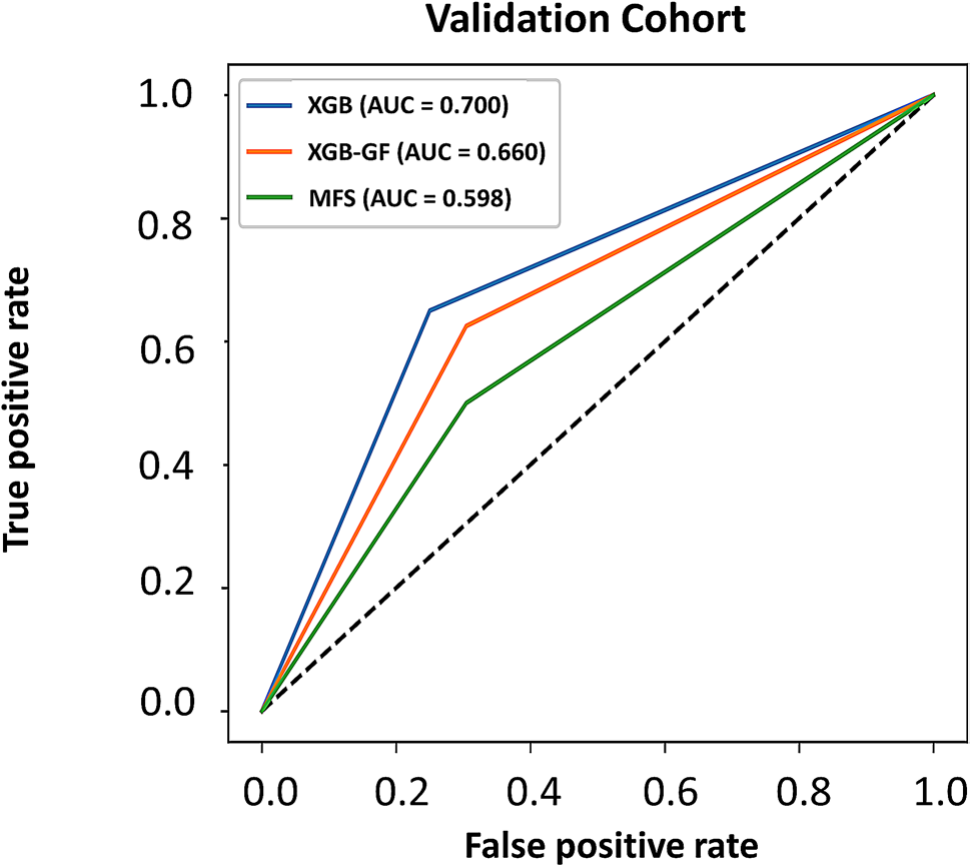
ROC curves for the MFS and XGB models.

Table 2 presents the contingency table for the prediction models. More than two-thirds of nonfallers were correctly identified by both the MFS and XGB (specificity: 69.6% and 75.0%, respectively), and more fallers were correctly identified by the XGB algorithm (sensitivity: 50.0% and 65.0%, respectively). Figure 1 presents the ROC curves and areas under the curves (AUCs) to assess the overall validity of the tools. There was no significant difference between the AUCs of the MFS and XGB (AUCs: 0.598 and 0.700, respectively; *P* = 0.09). Table 3 presents the sensitivity, specificity, PPV, NPV, LR + , and LR − of the two fall risk assessment tools. For the MFS, a PPV of 4.8% (95% CI: 3.2 to 7.3%) and NPV of 97.8% (95% CI: 97.0 to 98.4%) were calculated at the optimal cut-off point of 45 points, which was the same as the definition used in the original study. For XGB, a PPV of 7.4% (95% CI: 5.0 to 10.9%) and NPV of 98.6% (95% CI: 97.8 to 99.1%) were calculated at the optimal cut-off value. The likelihood ratios confirmed that the results according to the fall risk classification tools differed by chance, and the results only improved the diagnostic accuracy restrictively.

**Table 3:** Performance analysis for the prediction model (n = 132).

Scale/model	MFS	XGB	XGB-GF
Cutoff	> 45	> 0.53	> 0.58
AUC (95% CI)	0.598 (0.509, 0.682)	0.700 (0.614, 0.777)	0.660 (0.571, 0.738)
Accuracy (%)	63.64	71.97	67.42
Sensitivity (95% CI)	50.00 (33.8, 66.2)	65.00 (48.3, 79.4)	62.50 (45.8, 77.3)
Specificity (95% CI)	69.57 (59.1, 78.7)	75.00 (64.9, 83.4)	69.57 (59.1, 78.7)
PPV (95% CI)	4.8 (3.2, 7.3)	7.4 (5.0, 10.9)	6.0 (4.1, 8.6)
NPV (95% CI)	97.8 (97.0, 98.4)	98.6 (97.8, 99.1)	98.4 (97.5, 98.9)
LR + (95% CI)	1.64 (1.1, 2.5)	2.60 (1.7, 4.0)	2.05 (1.4, 3.0)
LR- (95% CI)	0.72 (0.5, 1.0)	0.47 (0.3, 0.7)	0.54 (0.4, 0.8)

AUC, area under the curve; CI, confidence interval; LR + , positive likelihood ratio; LR − , negative likelihood ratio; NPV, negative predictive value; PPV, positive predictive value; XGB-GF, the XGB model using generalizable factors including intrinsic factors and predictors directly linked to the cause of a fall.

Compromised results were found by XGB using only generalizable factors. The AUC of 0.660, sensitivity of 62.5%, specificity of 69.6%, PPV of 6.0% (95% CI: 4.1 to 8.6%) and NPV of 98.4 (95% CI: 97.5 to 98.9%) were calculated at the optimal cut-off value. The + LR and -LR values were similar to that of the XGB using the whole feature set.

### Feature importance according to XGB

**Figure 2:**
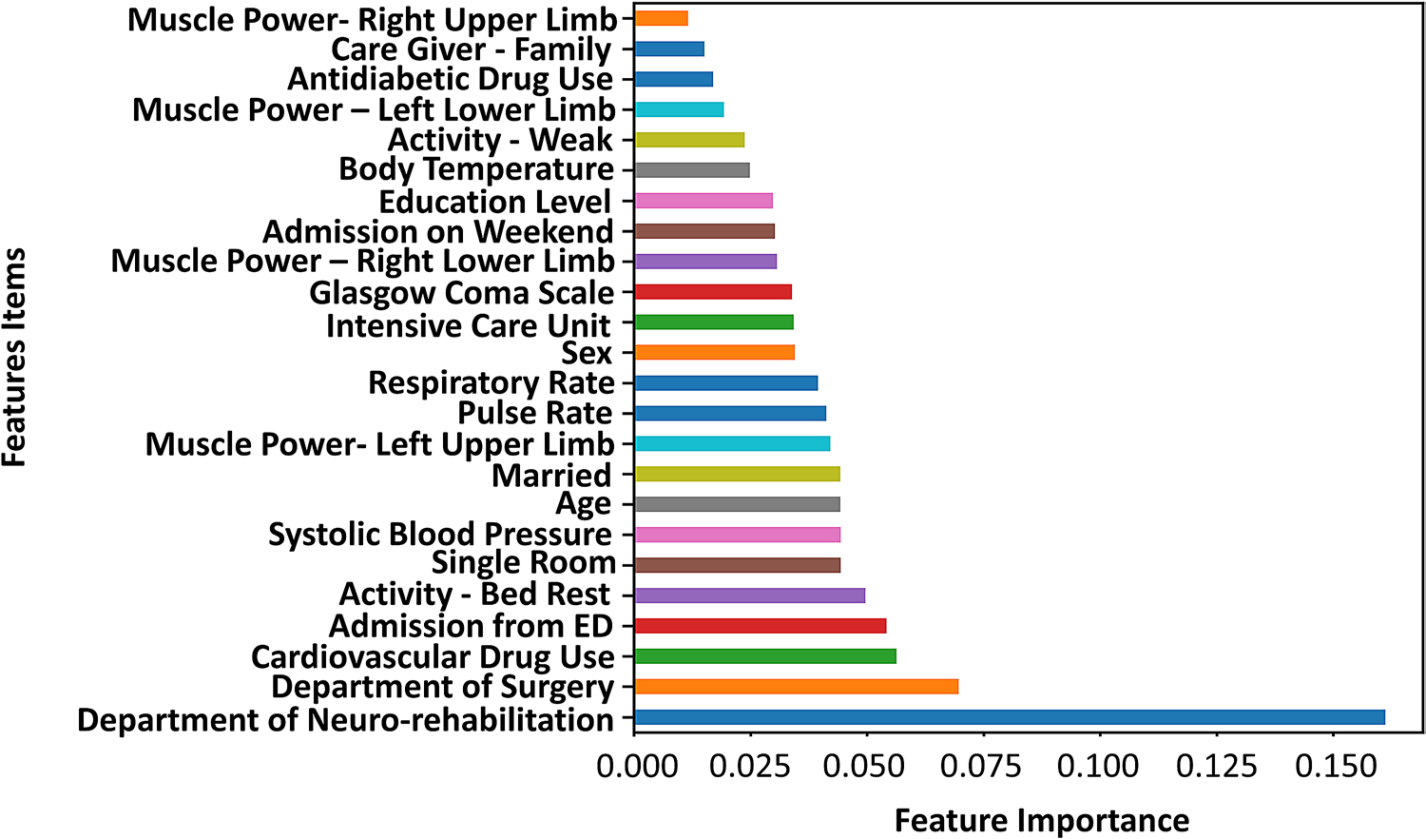
Feature importance of the XGB model.

Figure 2 shows the ranking of feature importance according to the XGB model. In this plot, the first column includes the item names of all the features that were actually used in the XGB algorithm, while each row lists the resulting importance scores calculated using the importance metric. The items were sorted bottom-up by the corresponding information gain values. The top five leading features, i.e., Department of Neuro-Rehabilitation, Department of Surgery, cardiovascular medication use, admission from the Emergency Department, and bed rest, are considered as the most important factors with a high impact on predicting inpatient fall events.

## Discussion

In the complex and complicated clinical scenario, both environmental factors and patient factors vary to a great degree. Currently, the most commonly used fall risk assessment tools, e.g., the Hendrich II Fall Risk Model, MFS, and the St. Thomas Risk Assessment Tool in Falling Elderly Inpatients (STRATIFY), incorporate only limited known risk factors that predispose a patient to fall risk^9–11^. The MFS consists of six evaluation items, and although patients are likely to fall due to a multitude of risk factors, such as postanesthetic weakness and unfamiliar environment, some of these factors are not assessed. In our study, the classification results showed that the MFS can identify approximately half (50.0%) of the patients who will suffer from a fall during their hospitalization. This result is similar to those of studies that have addressed the overoptimized results of the MFS^12^.

Although a single assessment using a rule-based assessment tool is simple and inexpensive, assessing patients continuously and following interventions outweigh the benefits. In our study, the XGB model took many risk factors for falls into account, and it identified approximately two thirds (65.0%) of the patients who suffered from a fall during their hospitalization. Among such patients, falls might be avoided if the patients are identified and effective preventive measures are taken in time. However, this potential benefit is countered by a low PPV (7.4%), which leaves much to be desired for using this approach. Despite involving a multifactorial computerization, the machine models to some extent suffer from time-dependent variables and confounding variables which are unstable and non-measurable during an inpatient stay. Furthermore, fall events show a stochastic trend. False-positive classification may result from the fact that predicted high fall risk is not necessarily associated with an actual fall event.

For this study was conducted in a single local hospital, efforts were made to validate this modality when generalizing the sample population and findings. The analysis of the feature subsets revealed that the effect of factors such as the department to which the patient has been admitted is probably not equally transferable to other hospitals. In general, the risks for falls are described as both intrinsic and extrinsic^13^. To better assess the generalizability of the approach, the XGB algorithm was run independently using only intrinsic factors and predictors that are directly linked to the cause of a fall. Comparing the results obtained from models over different feature sets, the XGB model using the full range of risk factors provided the best evidence for fall prediction. Although the performance of the XGB model was decreased when feature dimensionality was restricted to generalizable factors, it still substantially improved the identification of the fall-prone group.

Along with risk group identification, we have to recognize the most important predictors from the data sets and take precautionary measures to reduce the possibility of patient falls during hospitalization. An analysis of the feature importance of the items revealed that a number of factors associated with inpatient fall events are also found in the literature, as well as being a part of experiential clinical knowledge. First, extrinsic factors, including the Department of Neuro-Rehabilitation and the Department of Surgery, were associated with fall prediction in the XGB model. However, this result may be influenced by selection bias, as people are often admitted to certain units because they are physically handicapped and unable to live independently or are currently receiving postoperative care. There is a strong implication that fall events happen with greater frequency in certain areas where precautions should be focused. Second, the initial data mining from the medical records included a review of fall risk-increasing drugs (FRIDs), e.g., cardiovascular drugs, antidiabetic drugs, and CNS drugs, and the results showed that cardiovascular drugs were associated with a relatively higher risk for fall events. Our findings on cardiovascular drugs and fall risk are in conformity with the recent meta-analysis by de Vries et al. ^14^. Third, several intrinsic factors, including old age, unstable vital signs, musculoskeletal deficits and cognitive impairment, are conventionally regarded as risk factors and were confirmed by this study^15^. Despite our inherent assumptions, utilizing machine learning techniques for fall risk group identification can help prevent at least a certain percentage of falls as we correctly predict these events and take precautions based on the evidence.

There were several limitations to this study. First, classification models based on machine learning tend to be unstable in small datasets. Therefore, both models in this study were externally validated using a prospective cohort. Second, missing data for some subitems in the medical records confined the integrity of data mining; thus, only the fundamental items were selected for developing the model for fall risk classification. Third, PPV and NPV were influenced by the incidence of an event in the study population. The prevalence of inpatient falls being estimated as 3% is a rough estimate according to the data collected from the registration system of the patient safety committee, and is therefore arbitrary to some extent. Finally, this study was conducted in a single local hospital. A large-scale, prospective study is required to further explore the validity and reliability of these scales.

## Conclusions

This study proposes that the XGB classification model, which is more sensitive than the MFS, is more appropriate for assessing the fall risk in hospitalized patients. Furthermore, we identified several intrinsic and extrinsic risk factors that enhanced the ability to determine the underlying information on fall risk among the population. When relevant information is documented in regular medical records, XGB may better provide important insights for fall risk assessment compared to conventional rule-based criteria. The validity and reliability of prediction models based on machine learning must be carefully studied in further prospective, large cohort studies before they are used in clinical practice.

## Methods

### Study design and participants

**Figure 3:**
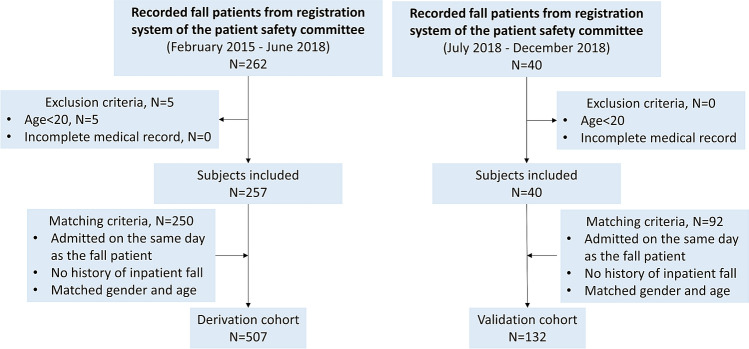
Flow diagram.

This study analyzed a cohort of patients hospitalized in Chiayi Chang Gung Memorial Hospital between February 2015 and December 2018 and was approved by the Institutional Review Board of Chang Gung Medical Foundation. Patients who experienced fall events were collected from the registration system of the patient safety committee. Patients who were under 20 years old and those who had incomplete data were excluded. For each fall case, up to three controls were randomly selected from the pool of patients who were admitted on the same day and had matched age and sex as the fall patient. Data were collected on the number of patients who fell rather than on the number of falls. All patients were retrospectively assessed for fall risk upon their admission according to the MFS. Basic patient information, medical records and demographic data were obtained. Figure 3 shows the flowchart of the study.

### Morse fall scale (MFS)

The MFS consists of six evaluation items, including a history of falling (0 or 15 points), secondary disease (0 or 15 points), ambulatory aid (0, 15, or 30 points), intravenous therapy/heparin lock (0 or 20 points), gait (0, 10, or 20 points), and mental status (0 or 15 points). The total score ranges from 0 to 125 points. A score below 25 is defined as the low-risk group, a score between 25 and 45 is defined as the intermediate-risk group, and a score above 45 is marked as the high-risk group^9^.

### Development of XGB

Chen et al. demonstrated the robust power of XGB system to control over-fitting in a variety of data mining challenges^16^. Furthermore, the classification tree structure of XGB is comprehensible and allows for the extraction of explicit classification determinants, which can be useful in risk stratification and subgrouping of the population. As a preliminary step, we tried to fit the training dataset to several types of models, including XGB, decision trees, random forest and linear discriminant analysis. The results also showed that XGB obtained the best performance in terms of sensitivity, specificity and AUC. Therefore, we have adopted XGB in this work.

Using data scraping techniques, 35 items were automatically extracted from standardized medical records, including the admission note and admission nursing record, and used for the data mining algorithms. The intrinsic factors and predictors which directly linked to the cause of a fall were defined as the generalizable factors. For both the whole feature set and a subset of generalizable factors, prediction models were trained independently using the training set and then benchmarked using the validation set. The items included in the extracted dataset are shown in Table 1.

The XGB, a scalable, supervised machine learning algorithm, is used to induce a classification model, as implemented in Python 3.4.3 with the XGBoost library.

To investigate the determinants for predicting inpatient fall events, feature importance was evaluated by using the information gain-based feature ranking algorithm. Information gain is a metric that quantifies the improvement in performance measure of a tree-based algorithm from each attribute that is split based on a given feature^17^. The information gain implies the relative contribution of the corresponding features to the model. A feature with a higher value in information gain among the whole feature set implies its significance for generating the prediction. Due to the inherent attribute selection process of the XGB algorithm, only a subset of the items actually appeared in the prediction model. A low-dimensional XGB model using a subset of features with high information gain was used to attempt to identify patients at a higher fall risk.

### Statistical analysis

Statistical analyses were performed using MedCalc 18.9.1 (MedCalc Software, Ostend, Belgium). Observed distributions were tested against the hypothesized normal distribution (Kolmogorov–Smirnov test). Data were reported as the mean ± standard deviation or number (%) unless otherwise indicated. The ROC curve is a plot of true positive rate against false positive rate evaluated at consecutive cutoff points of predicted probability. The area under the curve (AUC) measures the discriminatory ability of a model, where a value of 1.0 indicates perfect discriminatory power and a value of 0.5 indicates no discriminatory ability. To determine and compare the discrimination power of the MFS and XGB, the sensitivity and specificity were analyzed based on the area under the receiver operating characteristic (AUC-ROC) curve analyses. The optimal cut-off values for the ROC curves were determined using a maximized Youden’s index^18^. ROC curves were compared using the method described by DeLong et al. ^19^. The classification results for each model was summarized by a 2 × 2 contingency table, and the performance was assessed by calculating sensitivity, specificity, positive predictive value (PPV), negative predictive value (NPV), positive likelihood ratio (LR +), and negative likelihood ratio (LR–) ^20^. The PPV and NPV were calculated with an estimated incidence of 3% according to the data collected from the registration system of the patient safety committee. In all analyses, *P* < 0.05 indicates statistical significance.

### Ethics approval and consent to participate

The study was approved by the Institutional Review Board (IRB) of Chang Gung Medical Foundation, in accordance with the ethical standards of the responsible committee on human experimentation (IRB Nos. 201900460B0). Informed consent was obtained from all study participants in the manuscript.

### Consent for publication

Not required.

## Data Availability

All data generated or analyzed during this study are included in this published article.
